# The relationship between birth season and early childhood development: Evidence from northwest rural China

**DOI:** 10.1371/journal.pone.0205281

**Published:** 2018-10-11

**Authors:** Yu Bai, Guanminjia Shang, Lei Wang, Yonglei Sun, Annie Osborn, Scott Rozelle

**Affiliations:** 1 Centre for Experimental Economics in Education (CEEE), Shaanxi Normal University, Xi’an, Shaanxi, China; 2 International Business School, Shaanxi Normal University, Xi’an, Shaanxi, China; 3 Rural Education Action Program (REAP), Freedom Spogli Institute for International Studies, Stanford University, Stanford, California, United States of America; Hamamatsu University School of Medicine, JAPAN

## Abstract

**Objective:**

To examine the correlation between birth season and early childhood development.

**Background:**

Almost all previous studies that examine the effect of birth season on early childhood development were conducted in developed countries with a limited sample size. The present study was conducted in poor, rural areas of western China, a developing region with a continental monsoon climate.

**Method:**

We administered a hemoglobin test to 650 infants (52% boys), aged 8–10 months, using a Hemocue Hb 201+ finger prick system, and assessed the cognitive and psychomotor development of sample infants using Bayley Scales of Infant Development.

**Results:**

Infants born in winter have higher Hb concentrations (*t* = 3.63, *p* < 0.001) compared to infants born in summer. Similarly, cognitive development scores (*t* = 5.17, *p* < 0.001) and psychomotor development scores (*t* = 10.60, *p* < 0.001) were significantly higher among winter-born infants.

**Conclusion:**

The findings point to the involvement of birth season in early childhood development and suggest that aspects of the environment shape the experiences that contribute to early childhood development. Policy suggestions such as providing infants with ample opportunities for movement and stimulation during the cold season are discussed.

## Introduction

The first years of life comprise a critical development period that affects lifelong outcomes. This period has been identified as an important window for skill formation, especially for cognitive development [1]. Research on child development emphasizes the importance of the ‘first 1000 days,’ including the prenatal period and first two-plus years after birth; research is clear that the results of nutrition and stimulation deficits during this period are difficult to reverse [2–6]. Long-term follow-ups of experiments that increased investments in the nutrition, health, and stimulation of young children have shown positive consequences on later-life outcomes, including higher educational attainment, higher earnings, better adult health, and even lower participation in crime [3,6–10].

An increasing number of studies have shown that young children in developing countries experience more cognitive and psychomotor delays than young children in developed countries; they also have higher rates of anemia, indicating poor nutrition during the important 1000-day window [11,12].

Young children in rural China are no exception [13,14]. A recent study found that 49% of 6- to 18-month-olds from rural Shaanxi Province in northwest China were anaemic, and 53% of 30- to 36-months-olds were delayed either in cognitive or in psychomotor development [13]. Another study showed that 40% of 1- to 35-month-olds in rural China suffered from cognitive or physical development delay [15].

While many different factors may affect early childhood development, one significant factor is season of birth [16]. This factor has not been studied in a developing country context, but previous studies in developed countries, including Australia, Israel, and the US, have found that infants born in winter consistently have higher scores on cognitive tests, start to crawl earlier, and are taller and heavier compared to those who were born in summer [17–21]. Another study, also conducted in a developed country (USA), found that summer-born infants tend to be educationally disadvantaged when they are children [16,22].

Why do we observe this relationship between season of birth and development outcomes of infants? According to Bayley [23], infants born in winter (from December to February) experience one of their most critical stages of development (the period where they are around six months old) during the warmer months of summer. This means winter-born infants may have more opportunities to crawl on the floor, wear lighter clothes that expose them to more stimulation, and enjoy longer-lasting outdoor activities [17,20,24,25]. Their mothers also may be more willing to bring infants outside for additional activities, which could stimulate infant cognitive development [17]. More time spent outdoors during summer months also may mean that babies are exposed to higher levels of ultraviolet radiation, which is known to be associated with high levels of vitamin D and positive outcomes in early child development [20,26]. Being born in winter further means that at 6 (or so) months, infants would have better access to high-nutrient seasonal food products, like fresh fruit and vegetables [27].

While previous studies have made important contributions in demonstrating the importance of the birth season, there are some weaknesses in the existing research. Many researchers have often only studied a limited number of outcomes linked to birth season. For example, Atun-Einy [17] examined only motor development, and Angirst & Krueger [16] looked only at educational achievement of children when they were school-aged children. McGrath [20] only examined anthropometry outcomes. In addition, almost all of the work investigating season of birth and development of young children has been conducted in developed countries [16,17,20,21]. There has been no relevant research, to the best of our knowledge, conducted in poor rural China, one of the most populous developing regions in the world. In fact, there are reasons to believe that studying this problem in the context of a developing country may be particularly important since families in such countries are almost certainly less flexible in terms being able to make adjustments in how their children are raised when the environment turns dark and cold in the winter (for the sake of 6-month-old, summer-born infants).

In this paper, we examine the relationship between season of birth and early child development by using a unique dataset of infants drawn a developing county setting in northwest rural China. Unlike previous research, this is a large scale study conducted in a developing country setting. We hypothesize that, compared to summer-born infants, winter-born infants will have lower rates of anemia and higher scores on cognitive and psychomotor development scales. We further hypothesize that caregivers of summer-born infants will take their charges outside less frequently, feed them more limited diets, and bundle them in more restrictive clothing than caregivers of winter-born infants.

The rest of our paper is organized as follows. In the next section, we describe our research design, sampling methods, and statistical analyses. In the third section, we describe our results. The final section discusses and concludes.

## Methods

### Location

Our sample comes from 11 nationally-designated poverty counties located in poor rural Qinba mountainous area in the northwest of China. The overall climatic pattern of this part of China is characterized by two main seasons: a hot, humid summer and a cold, dry winter. In 2013, winter daily mean temperatures ranged from -2 to 16°C. Summer daily mean temperatures ranged from 18 to 38°C[28]. This continental monsoon climatic environment is similar to many places in the developed world (e.g., Haifa, Israel, the location of a study on birth season and early childhood development by Atun-Einy [17]); and to developing countries such as Bolivia and Mongolia, as well as high elevation regions of Northern Pakistan and Northern India [29–32].

### Sample selection

In 2013, the research team undertook the research initiative in eleven counties that the national government designated as being “officially poor.” The counties were in the Qinba mountainous area. The research team selected all townships in the eleven study counties. A township is the administrative district that is between a county and a village. After selecting the townships, the researchers randomly selected a village from every one of the sample townships.

We conducted our study in two waves. The first wave took place in April 2013, the second wave 6 months later, in October 2013. In each wave, we surveyed rural families with infants aged from 8 to 10 months old. The infants of first wave were born from June to August 2012. The infants of second wave were born from December 2012 to February 2013. In this way, we created two identically random samples according to our definition of the birth season. The sample size of infants born from June to August 2012 is 308; the sample size of infants born from December 2012 to February 2013 is 342.

### Data collection

#### Anemia

We collected Hb concentrations from all participating infants and their mothers. Hb concentrations were measured onsite using a Hemocue Hb 201+ finger prick system. Anemia status was assessed based on finger blood analysis for Hb, following the internationally accepted standard. The WHO considers children in our sample age range to be anaemic if their Hb concentration is less than 110 g/L [33]; moderate anemia is defined as Hb>70 g/L but < 100 g/L, and severe anemia is defined as Hb<70 g/L [34,35].

#### Cognitive and psychomotor development

In the study the enumeration team assessed the sample infants/toddlers using the Bayley Scales of Infant Development (BSID). In this manuscript, we use version-I of the Bayley Scales of Infant and Toddler Development. We use this version because in 2013, when this study was conducted, there were no Chinese versions of either Bayley-II or Bayley-III available. The BSID is a test that is scaled according to international norms. The scale was developed to analyze the cognitive and motor development of infants and toddlers [36]. To prepare the enumerators for the survey, the research team held a seven day long training program on executing the Bayles Scale. According the international protocol, researchers administer the BSID to infants according to a pre-set one-on-one format. The enumerators visit the household of each sample infant/toddler and conduct the assessment using a set of standardized toys and a pre-set scoring sheet.

The Bayles scale is one of the most frequently used assessment tool for assessing infant and toddler cognition and motor development. It is well recognized by researchers in the field of psychology. The American Psychiatric Association recognized it as a assessment tool that is able to diagnose certain developmental disorders [37,38]. Originally (in 1992), a national assessment team adapted the Bayles to the Chinese language/environment. It was scaled by repeatedly testing (norming) infants/toddlers in a normal, healthy urban Chinese sample [39–41]. In this paper, we build on the work on [15,42,43] that have utilized the Bayles scale in the study of infant development in China. According to the work of the norming team, both subsets of the Bayles scale (the MDI and PDI) have an inter-rate reliability of 0.99. Yi [40] has shown that the test-retest reliability rate also is high (0.82 for the MDI; 0.88 for the PDI). The same team also showed that the parallel forms reliability (0.85) was also high (0.85—MDI; and 0.87—PDI). These testing validity rates indicate that the test scores are reliable (consistent) even when there is variation in the methods used to give the test.

When assessing the level of development of an infant/toddler with the BSID, the protocol considers the age (in days/months) of each sample child. Additional consideration is given to children if they were born premature. When the study team considers these 3 factors, and assesses them in conjunction with the performance of the sample child on a series of tasks (using a standardized toy kit), the team is able to produce 2 standardized scores: the MDI, which evaluates memory, habituation, problem solving, early number concepts, generalization, classification, vocalizations, and language to produce a measure of cognitive development; and PDI, which evaluates gross muscle groups (rolling, crawling and jumping). The testing team also is able to assess fine motor manipulation (and create the PDI measure—[44]).

A careful protocol was followed in giving both the MDI and PDI parts of the Bayles protocol. After scoring by the testing team, both the MDI and PDI have been re-scaled. The expected mean of the score is 100 and the standard deviation is 16. When examining the entire spectrum of scores, they can span a range from 50 to 150, according to Yi [40]. After the official scoring, “mild impairment” is defined as 70≤MDI<80 and 70≤PDI<80. When infants and toddlers score less than 70, they are classified as having either moderate or severe impairment (MDI<70 or PDI<70 [40]). According to the scoring protocol, if infants/toddler score less than 50, the are automatically assigned a score of 49 [45].

#### Infant and family characteristics

Trained enumerators collected socioeconomic information and parenting behaviors from all households participating in the study. Each child’s primary caregivers were identified (the primary caregiver was individually identified by each family as the individual most responsible for the infant’s care, typically the child’s mother or grandmother) and administered a detailed survey on individual and household background, including child characteristics (birth date, gender, whether the infant was premature, iron-rich food intake, time spent outdoors), the primary caregiver’s characteristics (age, educational level, parenting behavior), and household information (whether the family was receiving Minimum Living Standard Guarantee Payments). The birth date of the infant was confirmed by checking the birth certificate issued by the birth hospital.

### Ethical review

This study received ethical approval from the Stanford University Institutional Review Board (IRB) (Protocol ID 25734), and from the Sichuan University Ethical Review Board (Protocol ID 2013005–01). All participating caregivers gave their oral consent for both their own and their infant’s involvement in the study. Children who were found to have severe anemia were referred to the local hospital for treatment.

### Statistical analyses

Statistical analyses were performed using STATA 14.2. P-values below 0.05 were considered statistically significant. The following Ordinary Least Squares (OLS) regression model was used for multivariate analysis:

$${ECD}_{ic}=\alpha +\beta ∙{Season}_{ic}+\gamma ∙X_{ic}+\lambda ∙s_{c}+\varepsilon_{ict}$$

Where the dependent variable, *ECD*_*ic*_, indicates the Hb concentration, MDI score or PDI score of infant i in village c. *Season*_*ic*_ is the birth season of the infant, and *β* is the parameter of interest. The term *X*_*is*_ is a vector of covariates that are included to capture and control for the characteristics of infants and their households. In particular, we included the following variables as potential confounders in the multivariate analysis: *gender*, *age*, *whether the infant was premature*, *whether the infant’s mother was the primary caregiver*, *maternal educational level*, *maternal age*, and *whether the infant’s family received minimum living standard guarantee payments*. Other non-time varying county effects are captured by λ. We adjusted standard errors by clustering at village level.

## Results

We sampled 650 infants (52% boys) aged 8–10 months. According to our data, around 4.6% of the sample infants were premature. The mother was the primary caregiver for 85.9% of the sampled infants. The majority of the mothers (74.9%) had completed at least junior high school; half (49.7%) were over 25 years old. Nearly 21.9% of sample families reported receiving ‘minimum-living-standard guarantee payments,’ a form of government welfare available for China’s lowest income families. Summary statistics for our sample are presented in Table 1.

**Table 1:** Basic characteristics of sample infants (N = 650).

Characteristics	Frequency (n)	Percentage
Gender		
Male	337	51.85
Female	313	48.15
Infant age (months)		
8	264	40.62
9	257	39.54
10	129	19.85
Infant was premature		
No	620	95.38
Yes	30	4.62
Mother is primary caregiver		
No	92	14.15
Yes	558	85.85
Mother completed at least junior high school		
No	163	25.08
Yes	487	74.92
Maternal age (years)		
< = 25	327	50.31
> 25	323	49.69
Families receives Minimum Living Standard Guarantee		
No	508	78.15
Yes	142	21.85

Note: Data are presented as frequency and percentage for all infants.

Applying the classification used by McGrath [20] and Atun-Einy [17], we divided this sample into two groups according to the season of birth, summer or winter (summer births = 47%; winter births = 53%). Summer births occurred from June to August and winter-born babies had birthdays from December to February. There is no difference between the two groups in any of the following characteristics: gender (*t* = 0.89, *p* = 0.38), whether the infants are premature (*t* = 0.96, *p* = 0.33), the mother being the primary caregiver (*t* = 0.31, *p* = 0.78), maternal age (*t* = 1.09, *p* = 0.27), maternal educational level (*t* = 0.36, *p* = 0.72), or whether the family was receiving minimum living standard guarantee payments (*t* = 0.18, *p* = 0.86).

Our sample infants experienced high rates of anemia and experienced more cognitive or psychomotor delays in comparison to the urban population used to scale MDI and PDI scores (Table 2). Hb concentrations for our sample were normally distributed with a mean of 108.4 g/L. 50.6% of infants had Hb concentrations below 110 g/L, indicating anemia. The mean MDI score for the sample was 97.6, significantly lower than the expected mean of 100 (SD = 15.3, *p* < 0.001). The BSID test results show that 4.6% of infants had an MDI score below 70, classifying them as moderately or severely impaired in their cognitive development. Around 7.7% of infants had an MDI score between 70 and 80, which indicates mild cognitive impairment. In total, 12.3% had MDI scores lower than 80. The mean PDI score for the sample was 90.4, significantly lower than both the observed MDI score and the expected mean PDI score of 100 (SD = 15.3, *p* < 0.001). The data show that 10.0% of sample infants were moderately or severely impaired in their psychomotor development (PDI < 70) and 10.46% were mildly impaired (PDI score between 70 and 80). In total, 20.5% had PDI scores lower than 80.

**Table 2:** Hb concentration, cognitive and psychomotor development of sample infants (N = 650).

	Mean/Percentage	CI (95%)
Hb concentration, g/L	108.43 ± 12.72	[107.93, 108.93]
Anemia status		
Anemia prevalance (Hb < 110 g/L)	50.61 (329)	[49.5, 51.5]
Mild anemia (100 g/L ≤ Hb < 110 g/L)	28.92 (188)	[28.5, 29.3]
Moderate anemia (70 g/L ≤ Hb < 100 g/L)	21.23 (138)	[19.9, 22.5]
Severe anemia (Hb < 70 g/L)	0.46 (3)	[0.2, 0.6]
Mental Development Index (MDI)	97.55 ± 15.32	[96.95, 98.15]
Cognitive impairment		
Any (MDI < 80)	12.31 (80)	[10.6, 14.0]
Mild (70 ≤ MDI < 80)	7.69 (50)	[6.8, 8.6]
Moderate or severe (MDI < 70)	4.62 (30)	[2.6, 6.6]
Psychomotor Development Index (PDI)	90.37 ± 15.27	[88.6, 92.0]
Psychomotor impairment		
Any (PDI < 80)	20.46 (133)	[19.7, 21.3]
Mild (70 ≤ PDI < 80)	10.46 (68)	[9.8, 10.8]
Moderate or severe (PDI < 70)	10.00 (65)	[8.8, 11.2[

Note: Data are presented as mean ± SD or % (Number of sample infants) for categorical variables.

MDI, Mental Development Index; PDI, Psychomotor Development Index.

**Fig 1:**
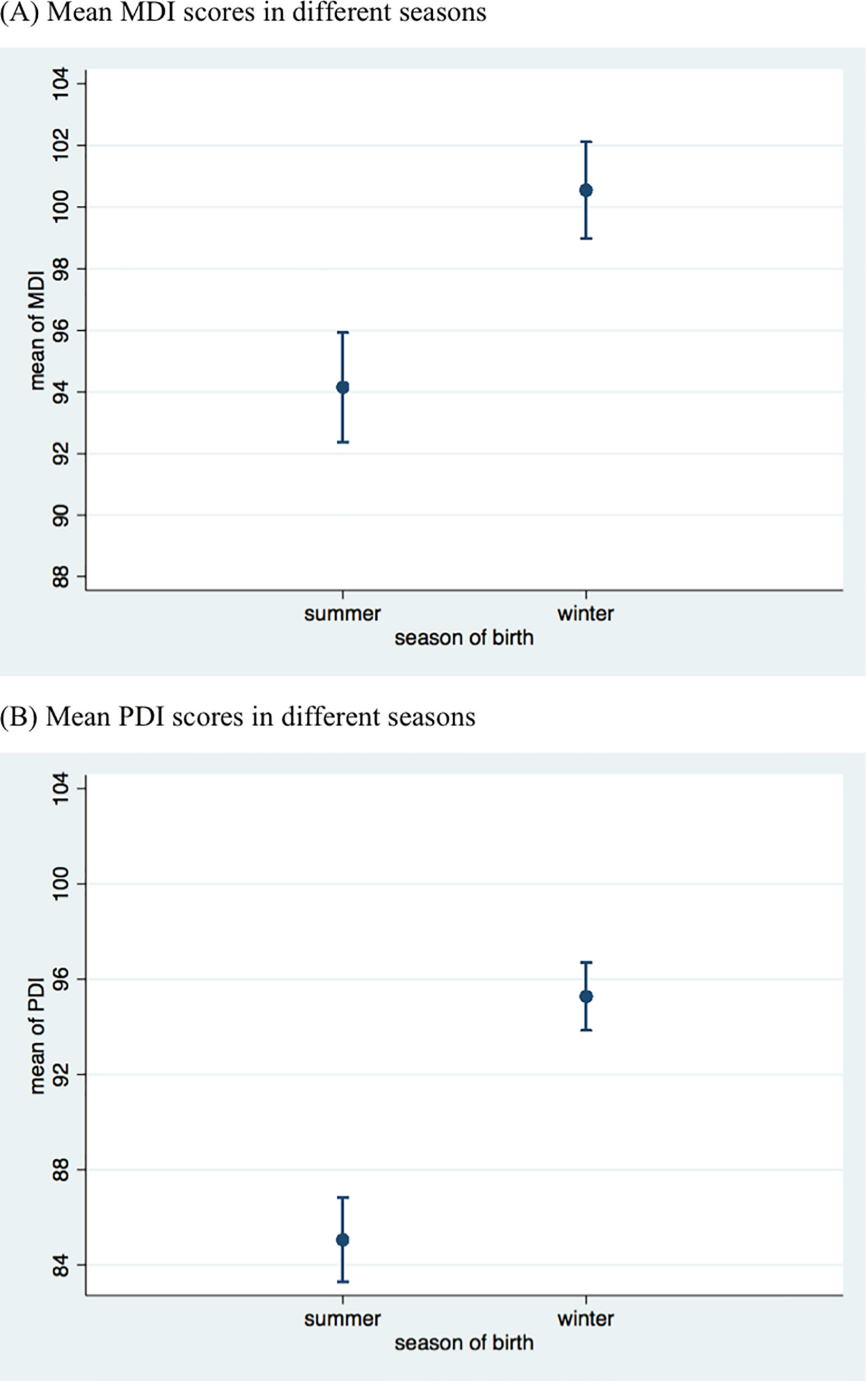
The status of cognitive and psychomotor development of infants in different birth seasons in poor rural Qinba mountainous area (N = 650). Panel A: Mean MDI scores in different seasons. Panel B: Mean PDI scores in different seasons.

The comparison of early childhood development outcomes between the winter-born and summer-born groups is presented in Table 3. Hb concentration was significantly higher in the winter-born group of infants, who reached an age of 6 months during the summer months (M = 110.7, SD = 12.1), than in the summer-born group (M = 105.9, SD = 12.9, *t* = 4.85, *p* < 0.001). In terms of MDI scores, we found that the winter-born group scored significantly higher on the MDI scale (M = 100.6, SD = 14.5) compared to infants in the summer-born group (M = 94.1, SD = 15.6, *t* = 5.49, *p* < 0.001). When comparing the PDI scores, we also found that the winter-born infants (M = 95.2, SD = 13.3) scored significantly higher than summer-born infants (M = 85.0, SD = 15.6, *t* = 9.09, *p* < 0.001). Similar results can be seen in Fig 1. In S1 Fig, we also display the average MDI and PDI scores for babies born in autumn and spring. As might be expected, the average scores lie between those of children born during the summer or winter.

**Table 3:** A comparison between the winter and summer groups: demographic and development status and caregiver behavior (N = 650).

Variables Frequency (SD)	Winter	Summer	*t*	*p*
	N = 342	N = 308		
Hb concentrations (g/L)	110.69 (12.10)	105.93 (12.94)	4.85	0.00
Mental Development Index (MDI)	100.58 (14.46)	94.12 (15.56)	5.49	0.00
Psychomotor Development Index (PDI)	95.24 (13.25)	84.97 (15.56)	9.09	0.00
Gender (1 = male; 0 = female)	0.46 (0.50)	0.50 (0.50)	0.89	0.38
Infant age (months)	8.96 (0.77)	8.61 (0.68)	6.15	0.00
Infant was premature (1 = yes; 0 = no)	0.06 (0.24)	0.03 (0.17)	0.96	0.33
Mother is primary caregiver (1 = yes; 0 = no)	0.85 (0.36)	0.86 (0.35)	0.31	0.78
Mother completed at least junior high school (1 = yes; 0 = no)	0.76 (0.43)	0.74 (0.44)	0.36	0.72
Maternal age (1 = more than 25 years; 0 = else)	0.48 (0.50)	0.52 (0.50)	1.09	0.27
Families receives Minimum Living Standard Guarantee (1 = yes; 0 = no)	0.22 (0.42)	0.22 (0.41)	0.18	0.86
Iron-rich food intake (1 = yes; 0 = no)	0.78 (0.41)	0.63 (0.48)	4.36	0.00
Time spent outdoor (hours)	1.78 (3.67)	1.25 (2.22)	2.21	0.03

Notes: Data are presented as mean SD for all children.

In terms of parenting behavior, our analysis also found significant differences between the winter- and summer-born groups. Infants born in winter were fed more iron-rich foods (including green vegetables, meat, and fruit) when they were 6 months old (*t* = 4.36, *p* = 0.00) and were taken outdoors more frequently by caregivers compared to summer-born infants (*t* = 2.21, *p* = 0.03).

The multivariate analyses show a significant association between season of birth and cognitive and psychomotor development after controlling for potential confounding variables (Table 4). More precisely, our analysis shows that being born in winter is associated with a 3.6 g/L (*t* = 3.63, *p* < 0.001) rise in Hb concentration, a 7.1-point rise in MDI score (*t* = 5.17, *p* < 0.001) and a 12.2-point rise in PDI score (*t* = 10.60, *p* < 0.001). No differences were found between the groups in other aspects, including infant gender, whether the infant was premature, whether the mother was the primary caregiver, maternal age, maternal educational level, or whether the family received Minimum Living Standard Guarantee Payments.

**Table 4:** Association of season of birth and development outcomes of infants in poor rural Qinba mountainous area (*N* = 650).

Variables	Hb	MDI	PDI
Winter born infant (1 = yes; 0 = no)	3.63***	7.08***	12.19***
	(1.00)	(1.37)	(1.15)
Gender (1 = male; 0 = female)	0.76	2.94***	2.60**
	(0.89)	(1.03)	(1.04)
Infant age (month)	1.69**	-2.16***	-6.85***
	(0.69)	(0.81)	(0.73)
Infant was premature (1 = yes; 0 = no)	-2.94	-0.16	4.90***
	(2.93)	(3.26)	(1.74)
Mother is the primary caregiver (1 = yes; 0 = no)	-1.79	1.34	1.22
	(1.45)	(1.65)	(1.71)
Mother completed at least junior high school (1 = yes; 0 = no)	2.11	-0.96	0.67
	(1.31)	(1.53)	(1.41)
Maternal age (1 = more than 25 years old; 0 = else)	0.38	-0.71	-0.85
	(0.94)	(1.09)	(1.09)
Families receives Minimum Living Standard Guarantee (1 = yes; 0 = no)	0.22	-0.51	-2.00
	(1.19)	(1.32)	(1.34)

Note

*** p<0.01

** p<0.05

* p<0.1. Robust standard errors in parentheses clustered at village level.

## Discussion & conclusion

The current study examined the seasonality effect on early childhood development by comparing infants born in different seasons in poor rural Qinba mountainous area in China, which is characterized by a continental monsoon climate. This study represents the largest administrations of the Bayley Scales of Infant Development (BSID) ever conducted in rural communities. The data collected include a rich set of indicators on early childhood development and its relevant factors, including cognitive and psychomotor development, nutrition and health, and feeding behavior. This study is also the first, to the best of our knowledge, to investigate the relationship between season of birth and early childhood development in rural China.

Our findings of high anemia and developmental delay rates in rural Chinese infants are supported by previous literature. For instance, our finding that 50.6% of infants had a hemoglobin concentration below the 110 g/L cut-off is in line with the findings of Grantham-McGregor [11] and Luo [13], that half of rural Chinese infants are anaemic. We also found that 12.3% of infants aged 8–10 months were cognitively delayed and 20.5% of them experienced delayed psychomotor development. These findings are similar with those reported in Wei [46] that between 12–21% of 1- to 35-month-olds in rural China showed delays in communication, gross motor development, fine motor development, problem solving, and personal-social skills.

As predicted, we found that infants born during the winter months tended to have higher average Hb concentrations, MDI scores, and PDI scores compared to summer-born infants. Our analysis found no other significant differences in terms of gender, whether the infant was premature, who the primary caregiver was, maternal age, or maternal education level, indicating that birth season in fact had a causal effect on these results.

The role of climatic factors and seasonal changes in shaping experiences should be underlined as a potential explanation for the observed seasonal effect on early childhood development. For example, in cold weather, it is typically necessary to use many layers of clothing to bundle up infants, which can constrain their movement. Groenen, Kruijsen, Mulvey and Ulrich [47] maintain that wearing bulky clothes in infancy can constrain movement sufficiently to delay exploration and performance of motor skills. This same restrictive clothing may mean that infants are also not exposed to enough stimulation by touch, which can result in both cognitive and psychomotor delays.

The consequences of bundling infants are particularly relevant in poor rural Qinba mountainous area, where daily average temperatures in winter range from -2 to 16°C. Mothers and caregivers in this area often perceive this weather as ‘very cold,’ and dress their infants in multiple layers [48]. It is a common belief that inadequate clothing during the winter and exposure to the wind (even a breeze) can cause diseases among infants [49]. Additionally, houses in poor rural Qinba mountainous area typically use inefficient heating equipment in winter, meaning that the temperature often remains low even indoors [50]. This can further prevent summer-born infants, who reach 6 months of age in winter, from exploring and developing to their full potential.

However, during the hot summer, infants in poor rural Qinba mountainous area often wear very light clothing, and there are more opportunities for them to crawl on the (warm) floor. Caregivers are more willing to take their infants outdoors in summer, which means their infants are exposed to more stimulation. And indeed, our analysis found that infants born in winter were taken outdoors more frequently by caregivers than infants born in summer.

Another seasonal environmental factor that should be noted is the availability of iron-rich food (including meat and green vegetables) in summer. Winter-born infants are typically ready to begin complementary feeding in summer, when parents might be more able to provide them with iron-rich foods. This was also borne out by our results, which showed that winter-born infants typically ate more green vegetables, meats, and fruits at around 6 months of age than summer-born infants.

Finally, a third important environmental factor associated with season is sunlight, which has been identified by several international studies as a contributor to early childhood development. A study conducted in Finland found that activity level is linked to day length: longer days are associated with increased mean total day activity levels [51]. Interestingly, solar radiation was one of the main predictors of children’s emotional and behavioral states in day-care centers in Florence, Italy [52]. Daylight length variation has been suggested as one of the variables that may affect seasonal differences in growth [20]. Wake and sleep times, both important for early childhood development [53], are also influenced by sunlight [54]. A study conducted in Israel found seasonal effect on infant sleep regulation: in the summer months, sleep onset occurred at a later hour and more motor activity during sleep was detected, compared to winter [53]. In poor rural Qinba mountainous area, there is a significant gap in the sunshine duration between summer and winter. This could be one of the seasonal factors affecting infant development.

In this paper, our findings should be interpreted in the context of the following limitations. First, the analysis in this paper is limited by the relatively short-term follow-up period for the measurement of the MDI and PDI scores. It is possible that if we followed the children over time, the gap between summer-born group and winter-born group would gradually narrow. A paper by Tsuchiya et al. [55] did find that MDI and PDI score differences were smaller after a longer follow-up period. Second, we are not able to identify the casual relationship between Hb levels and PDI scores by birth season. This is an important correlation that calls for further research, because the causal mechanisms associated with different development among children with different birth seasons are essential to understand if policymakers are to effectively address the issue.

The results of this study, especially when viewed in the context of the international literature on birth season and early childhood development, suggest that there may be several ways for parents to guard against developmental delay in summer-born children. Parents should focus on providing infants with ample opportunities for movement and stimulation during the cold season (e.g. bringing them outdoors, letting them crawl on the floor). Additionally, parents should invest in heating methods that keep indoor temperatures suitably warm during winter, allowing their infants to move around more freely indoors and wear bulky clothes for a shorter amount of time. If heating their own houses is not feasible, parents should seek out other warm indoor areas where their children can move around unencumbered. For example, investments should be made in rural areas to build public parenting centers that provide warm, infant friendly spaces so that infants can experience more mobility and interaction with their environment even in cold weather.

## Supporting information

S1 Fig The status of cognitive and psychomotor development of infants in different birth seasons in poor rural Qinba mountainous area (N = 650). Panel A: Mean MDI scores in different seasons.
Panel B: Mean PDI scores in different seasons.
(TIFF)
