# Supplementary figures and images for: The relationship between birth season and early childhood development: Evidence from northwest rural China
Source: PLoS One. 2018 Oct 11;13(10):e0205281. doi: 10.1371/journal.pone.0205281 (PMC6181351; doi:10.1371/journal.pone.0205281)

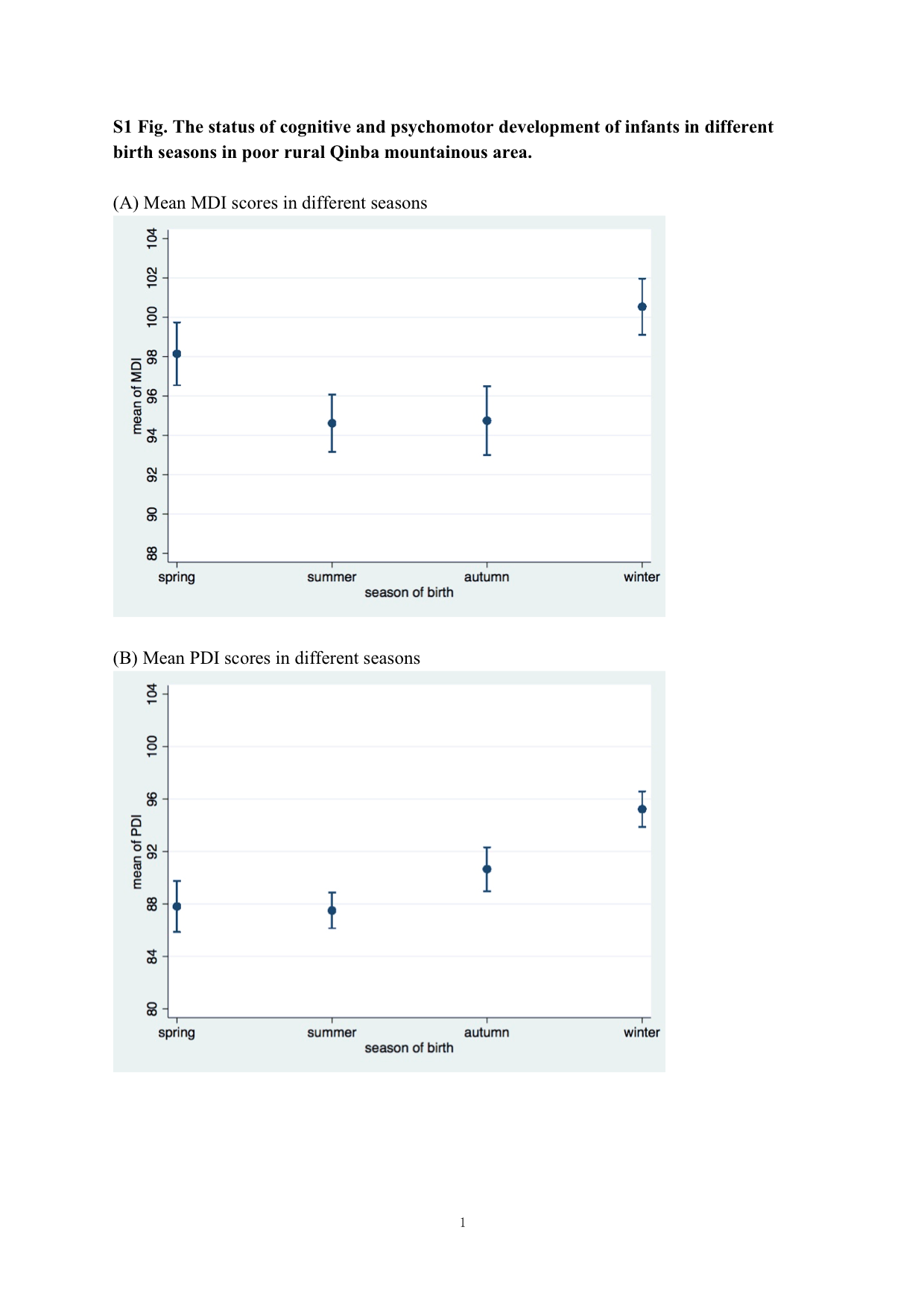

Supplement: S1 Fig — Panel A: Mean MDI scores in different seasons. Panel B: Mean PDI scores in different seasons. (TIFF) [file pone.0205281.s001.tiff]
